# Investigating Gendered Implicit Bias Among Healthcare Workers in South Africa: Development and Piloting of an Implicit Association Test in a High Tuberculosis and HIV Setting

**DOI:** 10.21203/rs.3.rs-9422562/v1

**Published:** 2026-06-17

**Authors:** Andrew Medina-Marino, Cho-Hee Shrader, Jordan Axt, Ernesha Webb Mazinyo, Kuhle Fiphaza, Nondumiso Ngcelwane, Singilizwe Moko, Joseph A. Daniels

**Affiliations:** Stellenbosch University; Rutgers, The State University of New Jersey; McGill University; Columbia University; Foundation for Professional Development; Eastern Cape Provincial Department of Health; Eastern Cape Provincial Department of Health; Arizona State University

**Keywords:** Tuberculosis, Implicit Bias, Explicit Bias, Men, South Africa, Health Services

## Abstract

**Background:**

In sub-Saharan Africa, men with HIV and/or tuberculosis (TB) report negative experiences within health services and demonstrate poorer engagement and treatment outcomes compared to women. We developed and piloted an Implicit Association Test (IAT) to assess healthcare workers’ (HCWs’) gender-based associations characterizing patients with the concepts of “good” versus “bad.”

**Methods:**

Standardized photographs of Black male and female faces from the Chicago Face Database were paired with locally derived descriptors of “good” and “bad” patients. The IAT was piloted among nurses providing TB or TB/HIV services in government clinics in Eastern Cape Province, South Africa. Following IAT administration, focus groups explored user experience and elicited discussions of “good” versus “bad” patients and perceptions of male and female patients. IAT data were analyzed using *D*-scores; focus group fieldnotes and artifacts were analyzed using a descriptive qualitative approach.

**Results:**

Eleven nurses participated and all completed the IAT, with 98% of trials retained after standard processing, indicating strong task engagement. Participants described the tool as understandable and usable, although some reported initial anxiety about responding “correctly.” The mean *D*-score was 0.234 (SD = 0.492), indicating a small relative association between male patients and negative attributes, though this association did not differ significantly from zero (95% CI: − 0.096, 0.565; *p* = 0.146). Qualitative findings revealed explicit characterizations of men as more likely to exhibit “bad” patient behaviors, aligning directionally with quantitative patterns.

**Conclusions:**

This pilot study demonstrates that a locally developed gender-based IAT can be feasibly administered among public-sector health workers in South Africa. Although the quantitative signal was small and not statistically significant, qualitative findings revealed explicit gendered evaluative perceptions, supporting the instrument’s internal face validity. This tool provides a contextually grounded approach to examining how gendered bias may shape health service delivery and establishes a foundation for larger-scale validation and implementation research.

## INTRODUCTION

Gender disparities in engagement across the HIV and tuberculosis (TB) care continuums remain a persistent challenge in sub-Saharan Africa. Globally, and in South Africa specifically, men living with HIV are significantly less likely to know their status, initiate antiretroviral therapy, or achieve viral suppression than women.^1–4^ Men also bear a disproportionate burden of TB disease, are less likely to optimally engage in care, and experience poorer treatment outcomes.^5–8^ Men frequently report negative experiences within health services, including unwelcoming clinical environments, negative staff attitudes, experiences of judgment and disrespect, and communication challenges with nurses.^9–13^ These experiences may reflect not only structural barriers but also underlying provider perceptions. Healthcare workers (HCWs) themselves have described men as “bad” patients—difflcult, ill-informed, prideful, or disengaged from care—characterizations that may shape the tone and quality of clinical interactions.^9^ While structural and social drivers of these disparities are increasingly recognized, far less attention has been paid to how healthcare workers’ perceptions of male patients may influence patient–provider interactions and engagement in care. Understanding and measuring such biases is therefore critical for designing gender-responsive HIV and TB service delivery strategies.

Gendered differences in care engagement have also been linked to hegemonic masculinity norms and broader social expectations regarding men’s strength, self-reliance, and help-seeking behaviors.^10,13,14^ However, less attention has been paid to how healthcare workers’ own perceptions of male patients may contribute to disparities in care experiences and outcomes. Given the documented sex differences in TB and HIV treatment outcomes^3,4,15–19^ and men’s reports of stigmatizing or dismissive treatment within health services,^9,10^ it is critical to examine whether and how bias may be operating within clinical environments in ways that exacerbate inequities in care delivery and outcomes.

Implicit bias, or “automatically invoked mental associations about social groups” occurs when an individual possesses an association between a group attribute (e.g., gender, race) and either a negative evaluation (i.e., prejudice) or negative category attribute (i.e., stereotype).^20^ Implicit bias is a form of structural discrimination impacting marginalized groups, manifesting based on historical, political, cultural, and societal norms and stereotypes.^20^ Implicit bias has been posited to alter a provider’s ability to form a therapeutic alliance with a patient, resulting in the provision of poorer health services and care.^21,22^ Although there is robust literature on implicit bias among healthcare workers (HCW) and its deleterious impacts on patient care experiences and health outcomes,^23,24^ little is known about implicit bias in healthcare settings in lower- and middle-income countries. Further, there is no identified research on how gender-specific implicit bias may impact the quality of care and patient health outcomes associated with TB or HIV.

Implicit association tests (IAT) allow for one means of measuring implicit bias.^25,26^ Among HCWs, most explorations of implicit bias using an IAT have sought to measure implicit racial or gender bias.^26–28^ Among IATs used in these studies, stimuli of target concepts have used White-Black faces, Asian-European faces, Light Skin-Dark Skin faces or Western-oriented Male-Female names, none of which are appropriate for an African context.^29–32^ To address this major gap, and explore gender-bias among South African HCWs, we developed and piloted an IAT using all Black Male-Black Female faces and evaluative characterizations of patients specifically as “good” or “bad.”

## METHODS

### Study Design and Setting:

We conducted a mixed-methods pilot study to develop and evaluate the feasibility and acceptability of a contextually grounded Implicit Association Test (IAT) designed to assess associations between patient gender and evaluative characterizations of patients as “good” or “bad” among healthcare workers in public-sector primary care clinics. The study was conducted in Buffalo City Metro Health District (BCM-HD), Eastern Cape Province, South Africa, between November and December 2021. South Africa remains a high-burden setting for TB and HIV, with an estimated TB incidence of 583 per 100,000 population and HIV prevalence of 12.7% among adults aged ≥15 years in 2021.^33,34^ TB incidence is higher among men compared to women (719 vs 459 per 100,000), while HIV prevalence remains higher among women (21.5%) than men (12.2%). Following IAT administration, focus group discussions elicited reflections on the tool and explored gendered perceptions of “good” and “bad” patients, as well as experiences with male and female patients.

### IAT Development:

Development of the IAT followed an iterative, locally grounded process to ensure contextual relevance. First, a series of structured workshops were convened with six (N=6) research staff (n=3 females; n=3 males) based in the Eastern Cape Province of South Africa; staff cadres included field-based data collectors and recruiters, lay health counsellors, project managers and coordinators, and program support staff. During these workshops, participants generated and discussed words commonly used to describe patients perceived as engaging positively or negatively with health services. This process yielded an initial pool of descriptors reflecting locally salient notions of “good” and “bad” patients, which were subsequently reviewed, refined, and consolidated to remove redundancy and ensure conceptual clarity (Table 1). In parallel, standardized facial images representing the target concepts (male and female) were selected from the Chicago Face Database.^35^ From the available Black male and female faces, the same group of staff reviewed images to assess perceived similarity to patients typically encountered in South African public-sector clinics. Through group discussion and consensus, faces judged to be most contextually representative were included, resulting in a final set of six male and six female facial stimuli used in the IAT (Table 1). Together, this process ensured that both attribute categories and target concept stimuli were locally informed and appropriate for assessing gender-based implicit bias in this clinical context.

The finalized target concepts and attribute categories were subsequently programmed into a computerized Implicit Association Test; additional information about the targets and attributes can be found in Table 1. The task was developed using Inquisit Lab (Millisecond Software),^36^ following established IAT design conventions, including a standard seven-block structure, counterbalancing of category pairings, and timed stimulus presentation to assess response latencies.^37^ The IAT was administered on Wi-Fi–enabled tablet computers using Inquisit Lab, allowing for standardized delivery in clinic-based settings.

The Inquisit software began with a series of instructions on how to complete the IAT. Participants were then instructed to complete a series of seven blocks: a practice block with the target images (e.g., male and female), a practice block with the attribute words (e.g., good and bad patients), two blocks with compatible pairings of target images and attributes (e.g., male and “bad;” female and “good”), a block with the targets images reversed (e.g., male and female reversed on screen), then two blocks with incompatible pairings of target images and attributes (e.g., male and “good;” female and “bad”). The first compatible and incompatible tests consisted of 20 trials and the second compatible and incompatible tests consisted of 40 trials, respectively; inclusion of compatible and incompatible tests allowed for counterbalancing of key positioning and prevention of order effects.

### Participant Recruitment:

Nurses providing TB or TB/HIV services within government primary health care clinics in BCM-HD were invited by the BCM-HD TB Program Manager (co-author N.N.) to participate in one of three scheduled IAT pilot events and focus group discussions (FGDs). Participants were told about the study upon their arrival at the meeting venue. Written informed consent was attained prior to study activities, and all participants were provided lunch (~$10 value) for their participation.

Prior to administering the IAT, study staff provided standardized instructions on how to use the tablet computer interface and how to complete an IAT. After answering all questions related to task navigation, participants were administered the IAT in a private space within the meeting venue. Following completion of the IAT, participants engaged in a FGD which explored their experiences and perceptions of the IAT (e.g., usability of the software, experience taking the test, clarity of instructions, perceptions of facial stimuli) as well as HCW views of patients receiving TB or TB/HIV services. Discussions were guided by a semi-structured discussion guide (Supplemental File) and moderated by two facilitators with support from a note-taker. Given concerns about sharing views in a group setting with colleagues of differing roles, FGDs were not audio-recorded. Instead, detailed field notes were taken during and immediately after each discussion. To enhance data quality, facilitators and the note taker immediately debriefed, reviewed and expanded notes following each session.

### Data Processing and Analysis:

IAT data were processed and analyzed following best-practice guidelines described by Greenwald et al. (2022)^38^ and Rohner and Thoss (2019).^39^ Briefly, response latencies shorter than 300 ms or longer than 10,000 ms were excluded, and participants with 10% or more responses with latencies shorter than 300 ms were excluded. Incorrect responses were penalized by replacing their values with the block mean of correct trials and adding 600 ms to the latency. Standardized *D*-scores were computed; positive *D*-scores indicate stronger relative associations of male patients as “bad”, whereas negative *D*-scores indicate stronger relative associations of female patients as “bad”. *D*-scores were interpreted using conventional thresholds to characterize the magnitude of implicit bias.^37^ A one-sample t-test was used to assess whether observed *D*-scores differed significantly from a null value representing no implicit bias in responses. IAT response latency data were processed and analyzed using the R statistical environment.^40^

Qualitative data included detailed field notes from focus group discussions^41^ and written artifacts^42^ generated during group activities, including lists and visual groupings of words describing “good” and “bad” patients. These materials were reviewed collectively to support triangulation of emerging concepts. Data were analyzed using a descriptive approach of study notes and artifacts, prioritizing low-inference description of participants’ language and dominant patterns.^43^ Field notes were reviewed for familiarization, and recurring language, dominant patterns, and illustrative examples were identified. Related concepts were organized into descriptive categories through structured analytic discussions among study team members.^44^ Immediate post-session debriefing and expansion of notes enhanced analytic completeness. This approach prioritized participants’ own language and perspectives, allowing analytic categories to remain grounded in how healthcare workers described patient characteristics while minimizing reliance on researcher-imposed interpretations.

### Ethical review:

Ethics approval was obtained from the Human Research Ethics Committee of the Faculty of Health Sciences, University of Cape Town (Ref no.: 673/2019) with an institutional reliance agreement by Arizona State University.

## RESULTS

### IAT Feasibility and Data Quality

**Figure 1:**
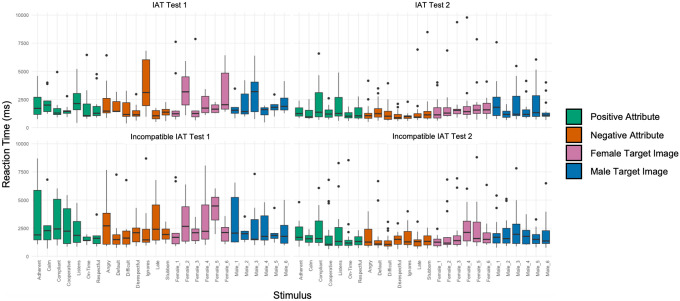
Mean latency (reaction time) distribution by stimulus

A total of eleven (N=11) nurses (n=7 female nurses; n=4 male nurses) consented to participate in study activities, all of whom completed all four critical IAT blocks, contributing 1,320 total trials. Of these trials, 0 (0.00%) had response latencies below 300 ms and 27 (2.0%) exceeded 10,000 ms. Of trials, 94 (7.12%) were incorrect responses and these responses were penalized by replacing their values with the block mean of correct trials and adding 600 ms to the latency. After applying exclusion criteria and data processing procedures, 1,293 trials (98.0%) across all eleven participants were retained for analysis. Examination of stimulus-level response latencies demonstrated variability across individual face stimuli (Figure 1), although no consistent pattern suggested systematic differences attributable to specific images.

### IAT Directionality and Magnitude

**Figure 2:**
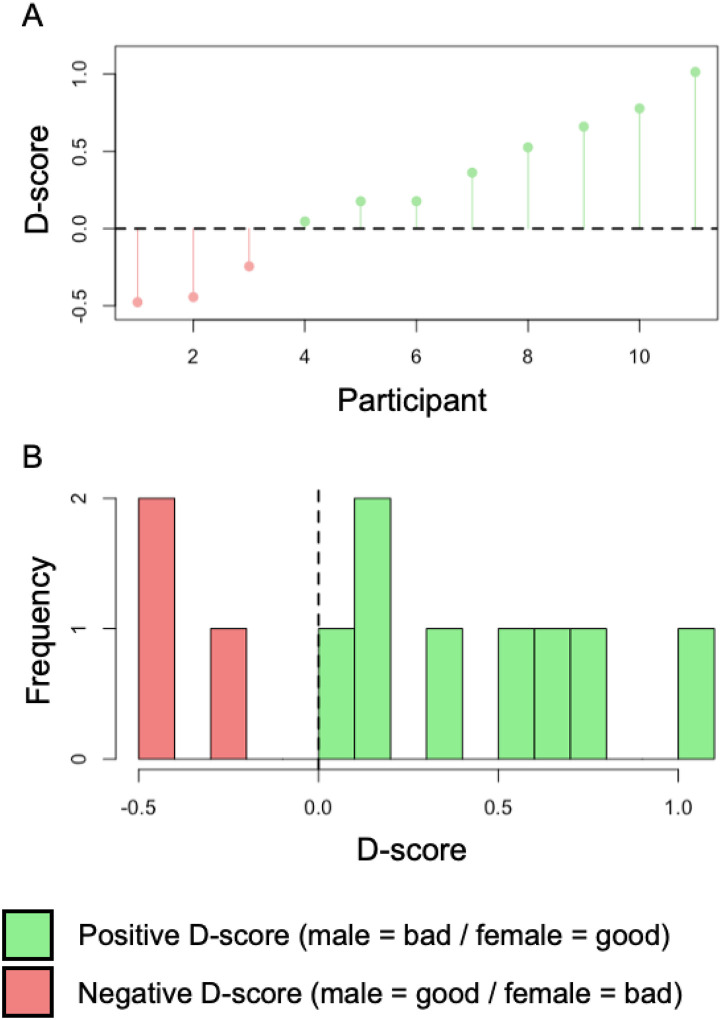
Lollipop plot (panel A) and histogram (panel B) demonstrating distribution of D-scores

The mean IAT *D*-score was 0.234 (SD=0.492, Cohen’s *d*=0.48), with individual scores ranging from −0.48 to 1.01 (Figure 2). Median IAT score was 0.177 (IQR: −0.099 – 0.593). Of the 11 participants included in analysis, 8 exhibited positive *D*-scores and 3 exhibited negative D-scores. A one-sample t-test comparing observed *D*-scores to a null value of zero (representing neutral implicit association) indicated that the mean D-score was not significantly different from zero (95% CI [−0.096, 0.565]; t(10)=1.578, *p*=0.146). Though the obtained effect size was consistent with a moderate association, the collected sample cannot rule out a neutral implicit association between male patients and negative patient attributes at the group level.

### Qualitative Assessment of IAT Acceptability and Feasibility

Three focus groups, consisting of 3–4 nurses each, were conducted. Participants’ overall reactions to the IAT were positive. Most participants agreed that the IAT and Inquisit Lab software were straightforward and easy to understand and use, though one participant reported not being experienced in using a tablet computer. Two participants described difflculty in understanding the IAT instructions, reported feeling pressure or fear of making errors, and that it took a while to get used to the IAT. A third participant reported laughing when she first saw the faces in the IAT and questioned herself, “*I wonder if I understand this correct?*” One group reported experiencing a network outage, resulting in a 40 min delay in conducting the IAT.

### Good Patient, Bad Patients

When asked to generate a list of characteristics of a “good” and “bad” patients for discussion, nurses described patients being adherent to their treatment, listened during patient education, picked up medication on time, individuals that stopped or did not smoke, were respectful to healthcare workers, and protected others from onward transmission of TB. These “good patient” attributes were predominantly ascribed to women and “she” pronouns were often used in describing “good” patients. One group of nurses described making additional efforts to care for patients perceived to be committed to their treatment, typically female patients, as those patients were respectful of their time and therapeutic efforts. Nurses ubiquitously described “bad” patients as those perceived as not being invested in their own health and TB treatment success. Behavioral characteristics of those individuals included using drugs, smoking, or abusing alcohol, as these behaviors undermined treatment adherence and cause patients “to default.” These behaviors were mainly ascribed to men, though younger patients were also described as engaging in “bad behaviors.” Patients with private medical care or government workers were described as having “attitude,” treated nurses with disrespect, and undermined nurses’ knowledge.

Nurses made a clear distinction between “bad” patients and those who were experiencing syndemic conditions that could impact TB treatment outcomes. In particular, nurses described how some patients with TB-HIV co-infection, particularly men, believed that they could only address one condition as a time.

Nurses also discussed how TB treatment could increase hunger, and how men, who already have “larger appetites,” were more likely to stop treatment due to “extreme hunger.” Patients with higher weight were also described as at-risk for poor adherence because of the increased pill burden associated with their weight. Other syndemic conditions eliciting empathy included lack of social support, unemployment, employment that interferes with clinic-attendance, poverty, lack of transportation, and poor integration of health service support (i.e., no social worker). Nurses noted that social support was uniquely important for men, older patients, and those living with HIV and/or experiencing poor mental health.

### Male Patient, Female Patient

Compared to women, men were generally perceived as being difflcult, “bad” patients due to non-compliance with TB testing procedures and engagement in care. Two focus groups discussed how men refused to provide sputum for testing or obtain an X-Ray and were more likely to only access testing services when they were very ill. In comparison, women were described as quicker to seek health care services before symptoms were severe. Men were also described as impatient, unwilling to navigate clinic wait times, and took issue when they could not see their preferred nurse (most often a woman). Taxi drivers in particular were described as “exceptionally impatient” because their time was linked to their salary. This caused some nurses to unfairly prioritize taxi drivers’ TB care above other patients. One group discussed how men would adhere to treatment until they felt better, and then “default” because they believed they were cured. Finally, nurses described male patients as “dishonest” because they would provide “false information” regarding HIV co-infection, smoking behavior, contact information, and treatment adherence. Female patients were generally described as being good listeners and honest about their treatment intentions, being active in their care regimen, attending appointments on time, and completing treatment. Participants described female patients as a mixture of being employed or unemployed, but also that they were “hustlers, unlike males.” One participant discussed how female patients were better patients than males; however, other participants clarified that this was true for older female patients only as younger female patients could also be “problematic.”

## DISCUSSION

To our knowledge, this is the first study to report the development and piloting of an IAT to assess associations between Black patient gender and evaluative characterizations of patients as “good” or “bad” in both the South African and the TB/HIV context. The administration of this new IAT among TB and TB/HIV nurses in South Africa was determined to be both quantitatively and qualitatively feasible. Although an implicit male-gender bias was identified among nurses, aligning with reported patient experiences in African settings,^10–13,45–47^ focus group discussions revealed explicit associations between men and qualities of “bad” patients. Development of this new IAT expands the tools available to explore how gender-based bias may be associated with and impact health outcomes, especially among men, and future uses of the measure may align with comparable IAT analyses that have shown correlations between individual IAT performance and systemic indicators of intergroup disparities or discrimination.^48,49,50^

Administration of the IAT was both operationally feasible and acceptable among public-sector nurses. All participants completed the IAT, and data quality indicators were consistent with established scoring guidelines, with the majority of trials retained for analysis after standard exclusions. Notably, no participants met exclusion criteria based on rapid response patterns (i.e., > 10% of trials with latencies < 300 ms), indicating sustained task engagement and adherence to established IAT data quality thresholds. Qualitative feedback further supported feasibility: most participants reported that the software was straightforward to use and instructions were clear, despite minor initial confusion and test-related anxiety among a few participants. Importantly, quantitative indicators of response latency and accuracy, together with participants’ reflections during FGDs, suggest that nurses were able to meaningfully engage with both the stimuli and task structure. Taken together, these findings support the feasibility of administering a gender-based IAT to health worker populations in South Africa.

In addition to feasibility, the pilot IAT produced a measurable directional pattern at the group level, with the mean D-score indicating a slight relative association between male patients and negative patient attributes. Although the magnitude of this association fell within the “small” range according to conventional interpretation thresholds, the pattern was observed among the majority of participants. However, the observed association did not differ statistically from zero, and the small sample size limits the precision with which group-level bias can be estimated. Importantly, this study was designed to pilot and assess the instrument rather than to definitively quantify population-level bias. As such, the magnitude and statistical uncertainty of the observed effect should be interpreted cautiously. Notably, the directional quantitative pattern aligned with qualitative findings in which participants explicitly characterized men as more likely to exhibit behaviors associated with “bad” patients. Together, these findings suggest that this locally developed IAT is capable of capturing gender-based evaluative associations among healthcare workers and may provide a useful tool for examining how such perceptions relate to healthcare workers’ beliefs, decision-making, and clinical interactions.

Findings from focus group discussions provided important contextualization and triangulation of the quantitative results. Nurses articulated clear and consistent associations between male patients and characteristics of “bad” patients, including non-adherence, impatience, dishonesty, and disengagement from care. In contrast, females were more frequently described as adherent, respectful, and engaged in treatment. These explicit characterizations mirror the directional pattern observed in the IAT results, strengthening the internal face validity of the instrument. Rather than identifying a discrepancy between implicit and explicit attitudes, our findings suggest that gendered evaluative associations may operate along a continuum that includes both automatic and consciously endorsed beliefs.^51,52^ The convergence of quantitative *D*-scores (although not significant) and qualitative narratives therefore supports the construct validity of the newly developed IAT and reinforces its potential utility for examining how gendered perceptions may shape health service delivery.

The gendered evaluative patterns observed in both quantitative and qualitative findings are unlikely to emerge in isolation from broader structural and cultural contexts. In South Africa, hegemonic masculinity remains a dominant social framework that positions men as strong, self-reliant, and economically productive, while stigmatizing vulnerability, illness, and help-seeking.^53^ Within such a context, men who present for TB or TB/HIV care may be perceived as deviating from expected masculine norms, potentially shaping how healthcare workers interpret their behaviors and engagement in care.^10,13^ These interpretations may reflect the internalization of widely circulating social narratives about masculinity, responsibility, and health rather than individual prejudice alone.^20^ Furthermore, health systems historically oriented toward maternal and child health services may unintentionally reinforce the perception of clinics as feminized spaces, contributing to gendered expectations about who is a “good” or “engaged” patient.^54^ Thus, the evaluative associations observed in this study may reflect structural gender norms embedded within both community and health system environments.^20^

Beyond broader gender norms, the qualitative findings highlight how health system constraints and syndemic conditions may shape perceptions of “good” and “bad” patients. Nurses distinguished between willful non-adherence and structural barriers affecting patient engagement, including HIV co-infection, food insecurity, unemployment, transportation challenges, and competing work demands. These findings align with prior qualitative research among South African men receiving TB care, which demonstrates how economic precarity, employment insecurity, and TB-related stigma interact to undermine sustained engagement in treatment.^10,13^ In resource-constrained public-sector settings, where clinic flow inefflciencies and limited appointment systems are well documented,^55,56^ behaviors such as impatience, missed visits, or delayed care-seeking may be interpreted as disengagement rather than as responses to structural pressures. These dynamics suggest that gendered evaluative patterns may emerge not only from cultural narratives of masculinity,^53^ but also from the interaction between patient-level constraints and system-level limitations. Addressing implicit or explicit bias, therefore, may require concurrent attention to service delivery redesign and structural supports that better accommodate men’s social and economic realities.

This study has several important strengths. The IAT was developed through a locally grounded, iterative process involving research staff familiar with public-sector clinic environments, enhancing the contextual validity of both the attribute categories and facial stimuli. Although images were drawn from the Chicago Face Database,^35^ local staff reviewed and selected faces perceived to be representative of Black patients commonly encountered in South African clinics, strengthening the relevance of the target stimuli. The mixed-methods design enabled triangulation of quantitative and qualitative findings, supporting internal face validity of the instrument. However, several limitations warrant consideration. The pilot sample was small and drawn from a single health district, limiting generalizability. Facial stimuli included only Black male and female faces and may not fully reflect the diversity of patients served in South African primary health care settings. This limitation may be particularly relevant in TB services, given the disproportionate burden of TB among Coloured communities with distinct phenotypic characteristics, potentially constraining TB-specific applications of the instrument. Focus group discussions were documented using detailed note-taking rather than audio recording, which may have limited nuance; however, structured facilitation and immediate debriefing supported collection of analytically rich data.^57^ Quantitative indicators of task completion and response latency further suggest meaningful participant engagement despite these constraints.

This study has important public health implications as it introduces a contextually grounded tool to examine how gendered evaluative biases within health systems may shape service delivery. Although originally developed to explore male gender bias in TB and TB/HIV services, where men experience poorer engagement and treatment outcomes compared to women,^8,56,58^ the instrument is not limited to assessing bias toward men and may be adapted to examine gender-based perceptions affecting both male and female patients across clinical contexts. While substantial evidence documents gender disparities in care experiences and outcomes,^54,59^ the contribution of healthcare worker bias to service quality, patient satisfaction, and downstream health outcomes remains insufflciently understood. By providing a standardized method to assess gendered evaluative associations, this IAT creates opportunities to empirically examine these relationships and to embed bias measurement within implementation and intervention research. Further validation and scale-up of this tool across diverse clinical contexts are warranted to examine how gendered evaluative perceptions may influence health service delivery and patient outcomes, and in turn support quality improvement initiatives to patient engagement in care.

## Supplementary Material

Table 1 is available in the Supplementary Files section.

Supplementary Files

This is a list of supplementary files associated with this preprint. Click to download.

- IATFGDGuideFINAL.pdf
- Table1.docx

## Data Availability

Upon written request to the senior authors (J.D. and A.M.-M.), data may be made available on a case-by-case basis.
